# Integrative, high-resolution analysis of single cells across experimental conditions with PARAFAC2

**DOI:** 10.1101/2024.07.29.605698

**Published:** 2024-07-30

**Authors:** Andrew Ramirez, Brian T. Orcutt-Jahns, Sean Pascoe, Armaan Abraham, Breanna Remigio, Nathaniel Thomas, Aaron S. Meyer

**Affiliations:** 1 Department of Bioengineering, University of California, Los Angeles (UCLA), CA, USA; 2 Department of Molecular Biosciences, Northwestern University, Evanston, IL, USA; 3 Computational and Systems Biology, UCLA, CA, USA; 4 Department of Computer Science, UCLA, CA, USA; 5 Jonsson Comprehensive Cancer Center, UCLA, CA, USA; 6 Eli and Edythe Broad Center of Regenerative Medicine and Stem Cell Research, UCLA, CA, USA

**Keywords:** PARAFAC2, tensor decomposition, single-cell analysis, scRNA-seq

## Abstract

Effective tools for exploration and analysis are needed to extract insights from large-scale single-cell measurement data. However, current techniques for handling single-cell studies performed across experimental conditions (e.g., samples, perturbations, or patients) require restrictive assumptions, lack flexibility, or do not adequately deconvolute condition-to-condition variation from cell-to-cell variation. Here, we report that the tensor decomposition method PARAFAC2 (Pf2) enables the dimensionality reduction of single-cell data across conditions. We demonstrate these benefits across two distinct contexts of single-cell RNA-sequencing (scRNA-seq) experiments of peripheral immune cells: pharmacologic drug perturbations and systemic lupus erythematosus (SLE) patient samples. By isolating relevant gene modules across cells and conditions, Pf2 enables straightforward associations of gene variation patterns across specific patients or perturbations while connecting each coordinated change to certain cells without pre-defining cell types. The theoretical grounding of Pf2 suggests a unified framework for many modeling tasks associated with single-cell data. Thus, Pf2 provides an intuitive universal dimensionality reduction approach for multi-sample single-cell studies across diverse biological contexts.

## INTRODUCTION

Single-cell measurements have revolutionized our ability to study the variation within and between heterogeneous cell populations^1,2^. As single-cell RNA-sequencing (scRNA-seq) and other high-dimensional single-cell techniques have become increasingly accessible, these technologies have extended to studies including multiple experimental conditions, samples, or subjects. Multi-condition single-cell experiments evaluate how heterogeneous cell populations behave in different biological environments, such as how cells respond to drugs and gene knockouts or how cells differ across patients with the same disease pathology^3^. However, there are substantial challenges in the analysis of these data, and appropriate analytical techniques will be essential to learning from these studies.

Given the size and complexity of single-cell data, mathematical frameworks are essential for deriving meaningful insights. Dimensionality reduction has been a central tool for exploration of single cell data, as it allows these large datasets to be summarized into patterns that are each associated across the cells and genes. Single cell experiments across conditions have three unique dimensions (cells, genes, conditions) that are often combined into one data matrix where each row corresponds to a cell, regardless of its condition, and the column represents a gene that is measured^4^. This process, where datasets with multiple dimensions are enforced to be two-dimensional, is known as flattening, resulting in conflating axes^5^. Because the cells and condition axes are confounded, the dimensionality reduction results are difficult to interpret because there is no distinction between how the cell and conditions contribute to this variation independently. Because of this challenge, other studies have employed pseudo-bulk techniques, wherein cells are coalesced into population averages, and differential expression is assessed for the corresponding population between conditions^6–8^.

Tailored computational methods have emerged to enable cell clustering, differential expression, gene set enrichment, and pseudotime analyses^9–12^. As single-cell datasets have grown to encompass multiple conditions, several techniques have been developed for the exploration and statistical analysis of these large datasets. For instance, one such method, PopAlign, leverages a few high-level cell types to align a reference and a test sample with a Gaussian mixture model^13^. Another method capable of modeling multi-condition data, PhEMD, identifies and aligns continuous variation in a cell state and is, therefore, most suitable for developmental or differentiation processes^14^. Other methods operate by contrasting two single-cell experiments: MELD uses graph signal processing to develop a relative likelihood estimate of observing each cell in each experimental condition to quantify the effect of a perturbation; HSS-LDA is a supervised dimensionality reduction algorithm that highlights differences between cell types based on a predefined annotation^15,16^. Loos et al. use a hierarchical modeling framework to compare varying levels of heterogeneity, ranging from cell-to-cell variability within subpopulations and across samples^17^. Finally, differentially abundant (DA)-seq identifies local clusters of cell states with differential representation^18^. However, these methods either make restrictive assumptions about how the data is distributed^13,14,17^; do not directly isolate patterns of variation to specific conditions, cell populations, and genes^14,15,18^; or reduce the analysis of single-cell variability to a specific number of pre-determined cell types^16^.

One solution to analyzing measurements across several dimensions is rearranging the data into a tensor, or a multidimensional array, and using a multilinear tensor decomposition technique^19^. One such tensor decomposition technique, canonical polyadic decomposition (CPD), approximates a dataset as the sum of the outer product of vectors, with each vector corresponding to a dimension of the data. For instance, CPD has been useful in bulk RNA-seq analysis measured across different tissues and patients; the three resulting component association vectors explain variance from each gene, tissue, and patient, respectively^20^. Where these methods are appropriate, we and others have observed several benefits of a tensor decomposition-based analytical approach^20–22^. Tensor decompositions can be more effective at removing noise, isolating distinct variation patterns, and imputing missing values compared to matrix-based techniques^20,23–25^. Most importantly, associating trends in the dataset to specific dimensions can help interpret the results and therefore derive insights from the underlying experiments^20,26,27^. Dimension-specific associations also make these methods especially effective in data integration for combining datasets with shared experimental parameters^20,28–32^.

Single cell experiments across conditions might be naturally organized into a three-dimensional tensor with axes representing the condition (e.g., samples, perturbations, or patients) in which the cells were collected, individual cells, and each gene measured. However, while conventional tensor decomposition techniques such as CPD assume alignment, or consistent correspondence across dimensions, single-cell technologies do not measure the same cell across conditions. One solution for decomposing data that lacks alignment along one dimension is an approach referred to as PARAFAC2 (Pf2)^33^. Pf2 derives a series of projections for each dataset onto a common reference frame. Originally developed to analyze data from chromatography experiments, where the elution time of molecules can vary from run to run, Pf2 has been applied in various contexts, including electronic medical health records and spectroscopy experiments^34–36^.

Here, we demonstrate that Pf2 provides an effective and general solution to integrating multi-condition single cell experiments by dissecting the variation found within and across conditions. Pf2 vastly improves the possible resolution of this analysis, identifying subpopulations of cells with distinct behavior that are missed by existing approaches. Pf2 is an unsupervised method that does not require external labeling of cells into cell types or categorization of the experimental conditions. Both benefits result in greatly enhanced associations with disease status in observational studies, and improved insights about the signatures associated with patient features. The well-developed theoretical foundations of Pf2 with connections to other tensor decompositions make Pf2 readily extensible to other challenges, such as integrating multi-omic or single-cell and bulk measurements. Based on these observations, we propose Pf2 as a broadly effective solution for analyzing single cell experiments across several samples or conditions.

## RESULTS

### PARAFAC2 integrates single-cell measurements across conditions through the principle of parallel proportional profiles

**Figure 1.**
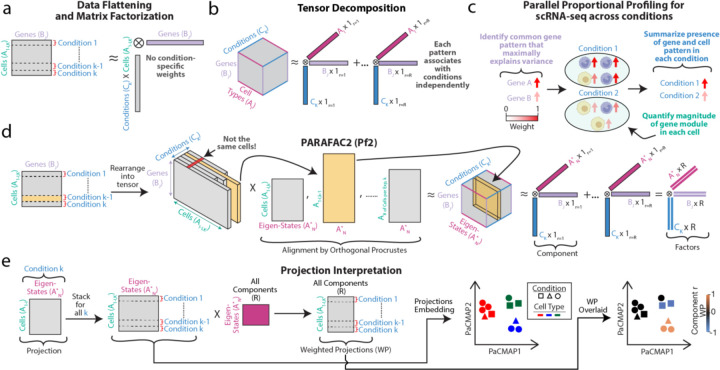
PARAFAC2 (Pf2) enables the interpretable integration of single-cell measurements across conditions. **(a)** Traditional data reduction methods are applied to flattened multi-condition data in matrix form. **(b)** Instead, tensor decomposition of aligned multi-condition data results in loadings with respect to each dimension. **(c)** Pf2 satisfies the principle of parallel proportional profiles which states that latent variable models can be most effectively aligned by identifying common underlying gene signatures in the dataset that only vary in their proportional magnitude within each condition or sample. **(d)** Multi-condition single-cell experiments can be naturally organized into a staggered three-dimensional tensor; Pf2 uses Orthogonal Procrustes to project cells onto common cell eigen-states and tensor decomposition (CANDECOMP/PARAFAC) to factor this aligned data. **(e)** The projections for each experimental condition multiplied by the eigen-state factors provide an association of each cell and component pattern. The projections alone also summarize the cell-to-cell variation independent of condition-specific effects.

Multi-condition single-cell data is typically analyzed by flattening/concatenation, where all single-cell experiments are combined into one large two-dimensional matrix of cells by genes (Fig. 1a). Consequently, once data reduction is performed on this matrix, the association of each component pattern with cells and conditions is confounded. Instead, one might imagine structuring this data as a condition by cell by gene tensor, and then applying CPD to create distinct component associations with each dimension of the single-cell dataset. While CPD is appropriate for data that can be arranged with corresponding cell types across samples (Fig. 1b), single cell data lacks the correspondence of the same or similar cells across samples^37^.

One solution for such data is PARFAC2 (Pf2); Pf2 and CPD is an implementation of Raymond Cattell’s 1944 principle of parallel proportional profiles, a set of rules for optimally aligning latent variable models across conditions (Fig. 1c)^33,38–40^. In the terms of a multi-condition single-cell study, a shared number of gene signature patterns are identified that maximally explain the variance across all experimental conditions.

The presence of a gene signature in each cell is quantified. Finally, the relative cell scores for each experimental condition are then summarized in their overall abundance for a condition. Pf2 introduces an additional constraint that enables Pf2 solutions are unique beyond trivial scaling and permutation differences, while avoiding restrictive assumptions such as non-negativity or a specific underlying cell distribution^13,38^. Most conservatively, Pf2 assumes similar variable cross-product matrices between experimental conditions, although many successful applications violate this assumption^38^. With scRNA-seq, this translates to the expectation of a roughly similar gene-to-gene covariance structure is similar across each condition^41^. Due to its application outside the biological sciences, the scaling and convergence performance of Pf2 has already been widely explored alongside additional constraints such as restriction to only non-negative factors^38,41–45^.

As part of its fitting process, Pf2 defines an aligned reference frame for the cells. This is accomplished by aligning the cells to each cell eigen-state by Orthogonal Procrustes (Fig. 1d). An eigen-state represents a weighted collection of cells with a shared set of gene signatures. This identifies common cells with shared expression profiles present across experimental conditions. The projections only contain information about cell identity, not condition-to-condition variation; they represent which cells are relevant when encompassing an eigen-state and map cells to an underlying latent space. Thus, the projections provide a common basis to compare cells with similar or dissimilar gene expression behavior across all samples.

After projecting the data into an aligned tensor, the projected tensor is factored via CPD^19^. CPD applied to the eigen-state intermediate tensor results in the weighted sum of rank-one vector outer products for the genes, eigen-states, and conditions. Similarly to PCA, each component, or a set of three vectors, describes the variation pattern associated to each dimension. Because each component comprises three separate loadings, each component isolates variation from specific genes, eigen-states and conditions. The combination of all vectors across one dimension are the factors and define how variation patterns vary across that one dimension. Because both the cell alignment and factorization are unknown, the two steps of cell alignment via the projections and CPD factorization are repeated until convergence (Fig. S1).

The Pf2 algorithm yields projections for each experimental condition and three factor matrices for the genes, eigen-states, and conditions. We adjusted the scaling, sign interpretation, and order of the components and eigen-states to improve factor interpretation (see in Methods). To determine which cells are highly associated with a specific component, the eigen-state factor matrix can be converted into a cell-specific factor matrix for each component (Fig. 1e). These “weighted projections” are derived by multiplying the eigen-state factor matrix and the projections across all conditions. By virtue of isolating the assignment of cells to each eigen-state, we can perform non-linear dimensionality reduction to visualize the projections in a low-dimensional embedding (Fig. 1e). This latent space describes cell-to-cell variation, separating any condition-specific effects. To illustrate which cells are associated with a given component, we can overlay the weighted projections on the projection embedding.

### Pf2 separates cell-to-cell from condition-specific variation

**Figure 2.**
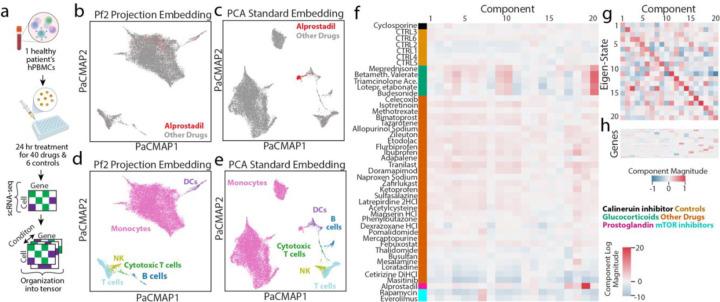
Pf2 defines cell-to-cell and drug-specific variation in a perturbational drug scRNA-seq experiment. **(a)** hPBMCs were treated with 40 FDA-approved drugs, including glucocorticoids, prostaglandins, mTOR inhibitors, and calcineurin inhibitors, alongside six controls. **(b–c)** PaCMAP embedding of the (b) Pf2 projections and (c) PCA-based dimensionality reduction, with cells from the alprostadil treatment condition highlighted. **(d–e)** PaCMAP of the (d) Pf2 projections and (e) PCA-based dimensionality reduction, with cells colored by their cell type annotations. **(f–g)** The (f) drug condition, (g) eigen-state, and (h) gene factors for each component. The genes factor matrix is filtered for genes having an effect magnitude of 0.8 or more within one of the components. The conditions factors are non-negative and have been log transformed to better show weights relative to the control conditions.

To explore the potential of Pf2, we first examined scRNA-seq data from a study wherein human peripheral blood mononuclear cells (hPBMCs) from one healthy donor were treated with a panel of 40 drugs from an FDA-approved immunomodulatory compound library, along with 6 control conditions (Fig. 2a)^13^. Previous analysis, using mixture modeling, isolated the transcriptional changes induced by each drug within two broad immune cell populations: T cells and monocytes (MO). Compounds were ranked based on their dissimilarity to the unperturbed controls to determine which experimental conditions most altered gene behavior. We surmised that Pf2 might improve on the resolution of the previous analysis by resolving smaller subpopulations with unique gene expression responses, as well as shared or distinct responses across drug perturbations. Pf2 and PCA explained less but similar amounts of variance using the same number of components, despite Pf2 being a more constrained model (Fig. S2a)^41^.

After fitting Pf2 with 20 components, we visualized the cell-to-cell variation using a PaCMAP embedding of the Pf2 projections (Figs. 2b,d)^46^. As a basis of comparison, we also plotted the PaCMAP embedding of the PCA scores with the same number of components (Figs. 2c,e). Whereas the PCA-based embedding resulted in clusters defined by their drug perturbation, the Pf2 projections-based embeddings distributed cells from each condition across the embedding space (Fig. 2b, 2c). Similar results were observed for other compounds (Fig. S2b–e). Manually annotating the cell types revealed that the Pf2 projection embeddings resolved many cell subpopulations despite condition-to-condition differences (Fig. 2d, e). To quantify these effects, we observed that the PCA standard embedding resulted in poorer integration accuracy in both batch integration and conservation of biological variance in immune cell types, most likely due to clustering cells based on perturbation-specific gene expression responses (Fig. S2f)^47^. As expected, given this experiment involves short-term treatment of PBMCs from one donor, abundant cell types were similarly represented across all conditions (Fig. S3a-g).

Like PCA, Pf2 results in a series of component patterns, but with weights representing the association of the pattern along the three dimensions of the data: drug perturbations (Fig. 2f), eigen-states (Fig. 2g), and genes (Fig. 2h). Each component pattern can be traced across the dimensions in the dataset and illustrates a distinct biological trend. A small fraction of genes was highly associated (i.e., highly weighted) with each component, consistent with our expectation that the effects of immunologic drugs on various cell types are defined through very specific gene signatures (Fig. 2h).

To determine whether Pf2 condition and gene factors trends were robust to changes in cell number, we applied Pf2 to subsets of the drug perturbation panel. Using the Factor Match Score (FMS) to quantitatively measure the similarity of the factor matrices, we found that the results were essentially unchanged with the dataset reduced to as much as half its original size (Fig. S4a)^45^. Using a bootstrapping strategy, we similarly observed consistent factor results at varying ranks, though a decreasing trend in the FMS at the highest ranks, as expected (Fig. S4b). These results indicated that Pf2 provides reproducible results and that we were not overfitting these data. With this in mind, we next wondered what biological insights Pf2 can identify.

### Pf2 identifies cell-to-cell, condition-specific, and hybrid gene expression variation

**Figure 3.**
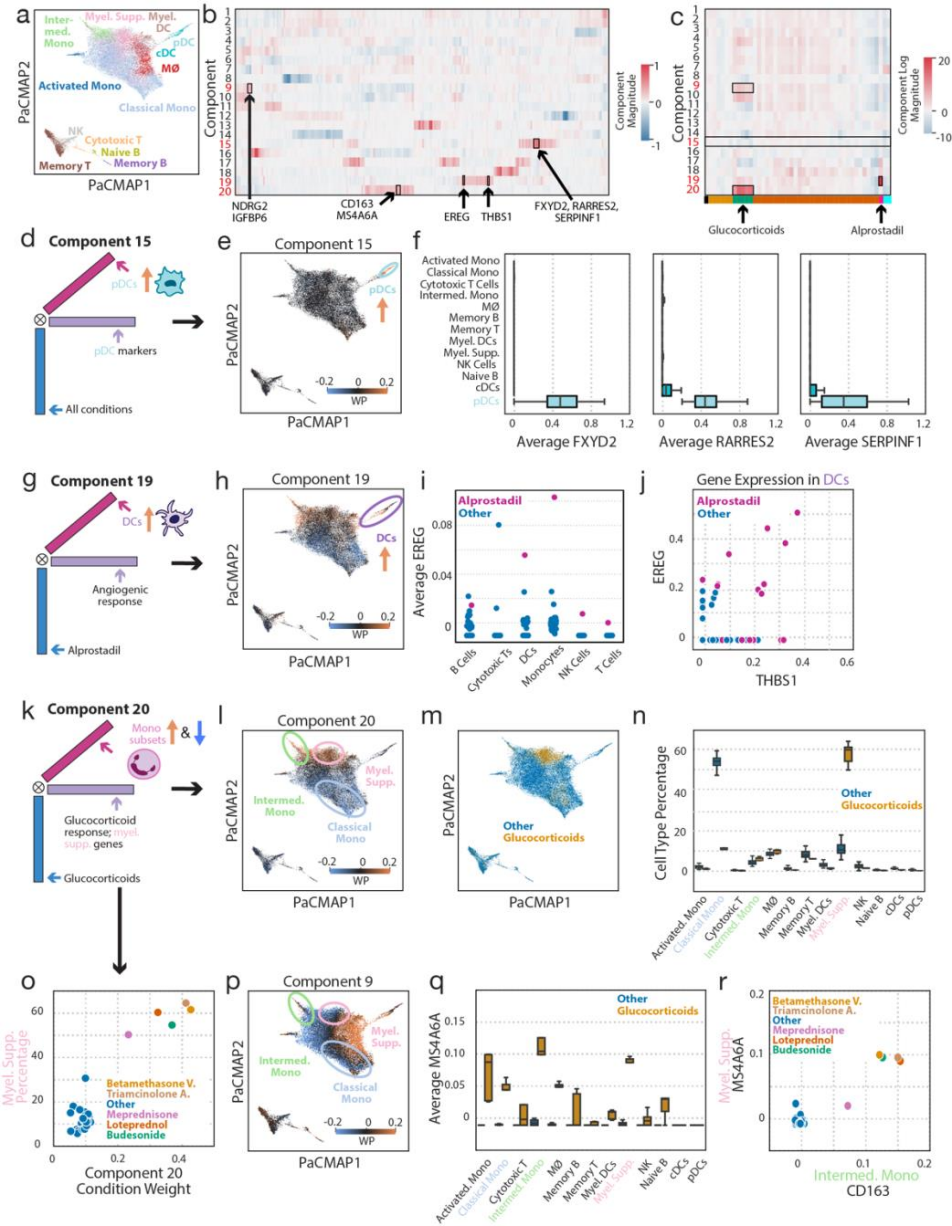
Pf2 analysis of drug perturbations reveals both broad and highly localized gene expression responses. **(a)** PaCMAP of the Pf2 projections, with cells colored by their high-resolution cell type annotations. **(b, c)** The (b) gene and (c) condition factors matrices with select genes and conditions highlighted. **(d, g, k)** Schematics of the biological significance of components (d) 15, (g) 19, and (k) 20 describing each components’ association with genes, eigen-states and conditions. **(e, h, l, p)** PaCMAP of the Pf2 projections, with cells colored by the weighted projections (WP) for components (e) 15, (h) 19, (l) 20, and (p) 9. **(f)** Per-condition average expression of FXYD2, RARRES2 and SERPINF1, stratified by cell type. **(i)** Average gene expression of EREG across drug conditions for low-resolution cell types. **(j)** Expression of THBS1 and EREG in individual cells, with alprostadil-treated cells labeled. **(m)** PaCMAP of all cells colored by whether they were treated with glucocorticoids. **(n)** Proportion of cells classified as each cell type for each condition, stratified by glucocorticoids versus all other drugs. **(o)** Percentage of cells classified as classical myeloid suppressors for each condition and their corresponding component 20 weight. **(q)** Average expression of MS4A6A within each cell type and stratified whether cells were treated with glucocorticoids or all other drugs. **(r)** Average CD163 expression in intermediate MO versus average MS4A6A expression in myeloid suppressors for each condition.

Having assessed the reliability of the Pf2 factorization, we next inspected the patterns of gene expression variation that were identified (Fig. 2f–h). To interpret the biological significance of a component, one can inspect the associations across specific conditions, genes, and cells. Immune cell types were further annotated to improve interpretation of the Pf2 components (Fig. 3a). One of the first advantages of Pf2 is the ability for components to identify common cells with similar gene expression profiles across experimental conditions. For instance, we plotted the component 15 weighted projections scores of individual cells on the PaCMAP embedding and found that plasmacytoid dendritic cells (pDCs) were selectively and highly weighted (Fig. 3e). FXYD2, SERPINF1, and RARRES2 are genes which have been identified as selectively expressed in pDCs in the literature and Human Protein Atlas^48–50^. These three genes were identified as the most strongly weighted in component 15, and further examination of these genes confirmed their specificity in pDCs (Fig. 3b). Examining the conditions factor, component 15 was largely invariant in its association with drugs, suggesting this component represents a gene expression module that is present across all conditions (Fig. 3c). Taking these findings together, we can conclude that component 15 represents the presence and unique gene expression of pDCs across all drug treatment conditions. Only 5 pDCs were found within each condition on average, demonstrating Pf2’s sensitivity to low-abundance cell populations (Fig. S5m). In addition to pDCs, Pf2 identified that component 12 was strongly associated with the amount of B cells in each drug perturbation experiment based on the genes identified by this component, including MS4A1A and CD79A (Fig. S6l, S7a, Table S2a). To simulate data where a cell population is differentially abundant in an experimental condition, we synthetically reduced or removed B cells from a drug perturbation experiment. The resulting Pf2 factorization had a reduced weight for component 10 in the condition factors, demonstrating how Pf2 identifies experimental conditions with differentially abundant cell populations within a condition (Fig. S7b, c).

In addition to isolating cell populations with consistent abundance across conditions, Pf2 also identifies patterns associated with specific drug perturbations. For example, component 19 was strongly and uniquely associated with alprostadil, a prostaglandin inducer of vasodilation and angiogenesis (Fig. 3c) ^51^. Component 19 most strongly weighted the DC population (Fig. 3h), and the component-associated genes included various ones relating to angiogenesis such as THBS1, VMO1, CXCL5, EREG and VEGFA (Table S2b). VEGFA and EREG have been previously confirmed to be upregulated in response to prostaglandin stimulation^52,53^. Alprostadil-treated DCs expressed the most EREG and THBS1, and were the only DCs to co-express these, demonstrating Pf2’s ability to identify coincident patterns of gene expression within individual populations (Fig. 3i, 3j). Thus, component 19 represents an angiogenic gene module expressed in DCs that is uniquely induced by alprostadil and illustrates Pf2’s ability to model condition- and cell population-specific expression patterns.

Pf2’s tensor-based structure can identify expression modules shared across several drug perturbations. For example, component 20 has a strong relationship with all the glucocorticoids, steroid hormones commonly used to treat inflammation, autoimmune diseases, and cancer with broad transcriptomic effects across immune cell populations (Fig. 2e)^13^. The variance encoded by component 20 is strongly represented in the monocyte population, with distinct effects by monocyte subtype (Fig. 3l). Here, intermediate MO and myeloid suppressors had positive weights, and classical MO (cMO) had negative weights, suggesting that the gene expression and abundance information encoded by component 20 is divergent across these subpopulations. The myeloid suppressor cluster cells were enriched in the glucocorticoid-based experiments, mirroring the cell specificity of component 20 (Fig. 3m). Indeed, when examining the cellular composition of samples treated with glucocorticoids, we observed a substantial decrease in cMOs, and an increase in myeloid suppressor cells (Fig. 3n). The distributions of all other cell types remained consistent across conditions, suggesting that our projections-based embedding was not simply being informed by drug perturbation, as with the PCA-based embedding (Fig. 2c). The component 20 weight positively correlated with the proportion of cells labeled as myeloid suppressors (Fig. 3o). This suggests that component 20 captures glucocorticoid-induced expansion of immunosuppressive phenotypes within the myeloid compartment^54–56^.

Although component 9 was also glucocorticoid-specific, it isolated distinct gene signatures and cell associations as compared to component 20 (Fig. 3c, p, Table S2a, S2b). Component 20 described glucocorticoid-induced CD163 and MS4A6A expression across cMO and other cell types, as seen in earlier studies (Fig. 3b, q, S7) ^57^. CD163 upregulation in intermediate MOs correlated with MS4A6A expression in myeloid suppressor cells (Fig. 3r), demonstrating Pf2’s ability to identify shared expression changes between populations. In total, Pf2 can identify both condition-specific expression patterns and cellular abundances differences, as demonstrated by its myeloid subtype analysis. However, interpretation of Pf2’s patterns must be investigated to determine whether a component represents cellular abundance of a cell type or cellular expression differences. Overall, Pf2 was able to effectively separate distinct and highly interpretable patterns of gene expression variation, whether cell type-specific (Fig. 3e/f), condition- and cell type-specific (Fig. 3h/i), or even conditions with shared alterations among subpopulations of cells (Fig. 3o–q). While we only investigated a select few patterns, the other components identified by Pf2 identify relevant biological insights as well (Table S1).

### Pf2 improves the resolution and accuracy of associations with systemic lupus erythematosus

With an understanding of the benefit Pf2 can provide within perturbational studies, we next examined its potential to separate cell-to-cell from patient-to-patient variation and be predictive of patient status within an observational cohort study. To do so, we focused on a recent study that characterized hPBMCs from a cohort of 162 systemic lupus erythematosus (SLE) patients and 99 healthy controls by scRNA-seq^58^. Multiple scRNA-seq samples were collected for some patients, resulting in 209 SLE and 145 healthy samples. Samples varied widely in overall cell number (Fig. S8g). The previous analysis relied on pseudobulk representation of several cell types to contrast patient groups^58^.

**Figure 4.**
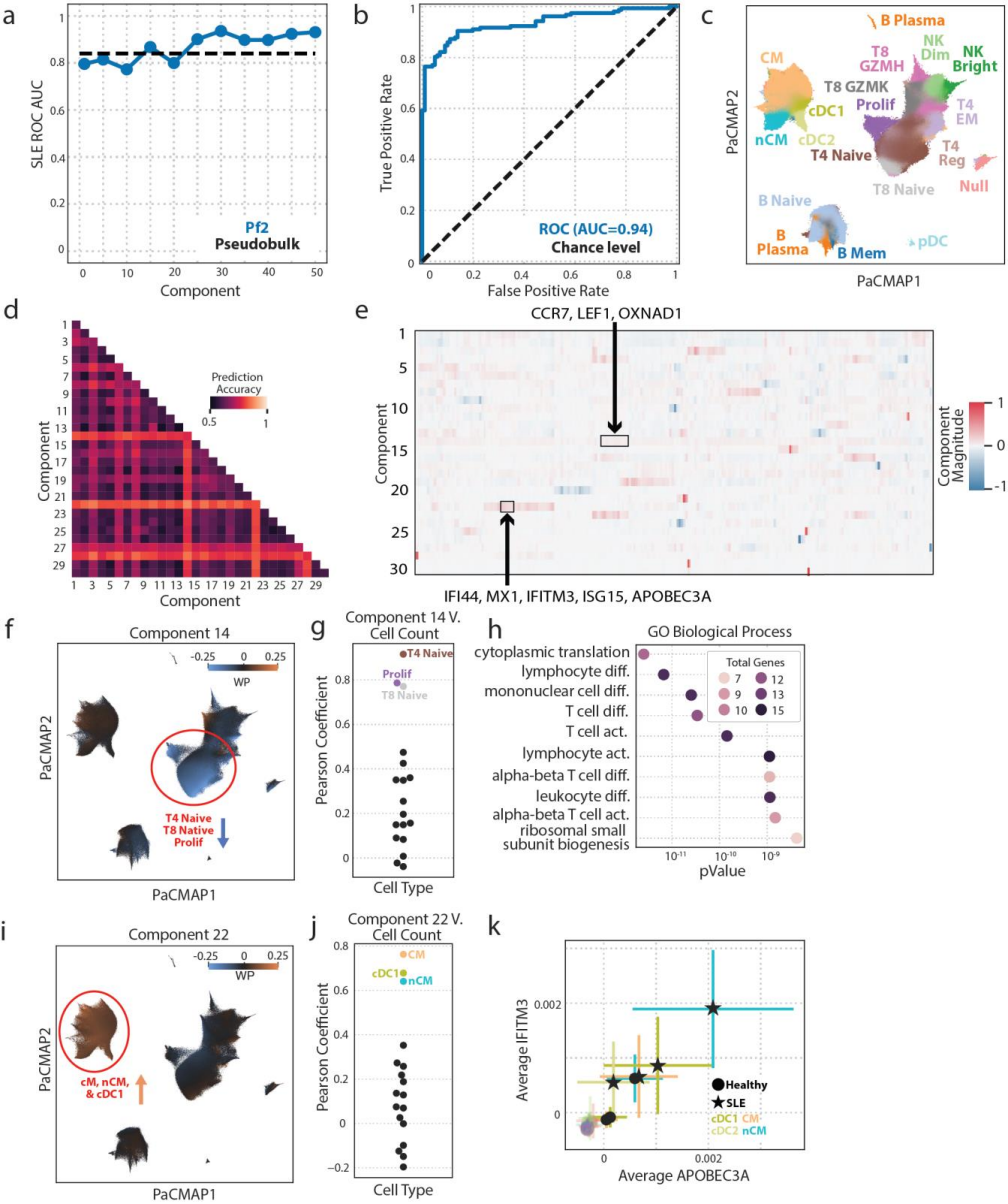
improves the resolution and accuracy of associations with systemic lupus erythematosus. **(a)** ROC AUC of a logistic regression classifier, using the patient factors, predicting SLE status. Pf2 rank was varied between each model. **(b)** ROC curve of a logistic regression classifier, using the patient factors, predicting SLE status with a 30-component Pf2 model. Cross-validation was performed by leave-one-processing-batch out. **(c)** PaCMAP of the Pf2 projections, with cells colored by their higher resolution cell type annotations. **(d)** For a 30-component Pf2 model, SLE prediction accuracy of an unpenalized logistic regression model trained on pairs of components. **(e)** The gene factors matrix using 30 components, with genes from components 14 and 22 highlighted. **(f, i)** PaCMAP of the Pf2 projections, with cells colored by the weighted projections of components (f) 14 and (i) 22. **(g, j)** Pearson correlation coefficient based on the relationship of cell count per patient against corresponding patient sample weightings of components (g) 14 and (i) 22. **(h)** Gene set enrichment analysis (GSEA) biological process results for component 14 gene module (top 30 positively weighted genes), where dot color represents the number of genes overlapping with the gene ontology. **(k)** Average expression of IFITM3 and APOBEC3A for low-resolution cell types in healthy and SLE samples.

We first investigated Pf2’s broad explanatory and associative power. Pf2 explained an increasing fraction of the dataset variance with more components (Fig. S9a). To select an appropriate number of components, we used a logistic regression (LR) classifier to associate the Pf2 patient sample (i.e., condition) factors with SLE status. Cross-validation was performed identically to the previous study, using batch-specific training and testing; briefly, we trained the LR model on one of the four processing batches of data and then used the patient sample factors to predict the status of the left-out samples^58^. Increasing the component number improved prediction accuracy for SLE status up to about 30 components, surpassing the accuracy of pseudo-bulk analysis (Fig. 4a, S9b). With an AUC ROC of 0.94 using Pf2, we continued using 30 components to represent the single-cell patterns present in the SLE cohort study (Fig. 4b). The 30 components were variously associated with the patients’ samples, eigen-states, and genes (Fig. S9c). Examining the cell-to-cell variation within the projections via the PaCMAP embedding, we observed effective separation of the expected immune cell types in hPBMCs, both at lower and higher resolution (Fig. S9d, 4c).

Using an LR model with an L1 penalty for the 30 patient factors, we identified which components were most relevant for differentiating SLE sample status (Fig. S9f). Even though the prediction accuracy was 0.98, L1 regularization eliminated multiple components that could possibly differentiate SLE sample status on its own. We therefore also assessed the classification accuracy using all pairs of components (Fig. 4d). Components 14, 22, 27, and 28 led to high classification accuracy in these reduced models. We again traced the components across the cell and gene dimensions to interpret the significance of each of these variation patterns picked out by Pf2 (Fig. 4e, Table S3a, b).

We first investigated component 14 as it was most strongly associated with SLE status. Component 14 selectively associated with Naïve CD4+ and CD8+ T cells, as well as Prolif, a subcategory of proliferating T cells (Fig. 4f). The sample associations of this component directly correlated with the cell abundances of these annotated lymphocyte populations (Fig. 4g). Consistent with explaining the abundance of these cell populations, the gene associations of component 14, including CCR7, LEF1, and PRKCǪ-AS1 (Fig. 4e), have been previously characterized as crucial for early T cell development, cell identity, and T cell receptor complexes^59,60^. Gene set enrichment analysis (GSEA) of the 30 most weighted genes for component 14 confirmed an association with T cell activation and lymphocyte differentiation (Fig. 4h, Table S3b).

Component 22 was also associated with SLE status and corresponded to the myeloid populations of CM, nCM and cDC1 (Fig. 4i). Similarly to the T cell development pattern, component 22 correlated with the cell abundance of these specific myeloid populations (Fig. 4j). This component was associated with interferon (IFN)-induced genes, including IFI44, MX1, IFITM3, APOBEC3A, and ISG15 (Fig, 4e). Other components overlap in these IFN genes, such as component 6, but highly weight distinct cell populations (Table S3a, b, Table S3). The IFN-stimulated cell types identified by component 22 uniquely express IFITM3 and APOBEC3A in combination (Fig. 4k). Thus, Pf2 recovered several known alterations associated with SLE^58,61^.

### Pf2 identifies novel single-cell patterns associated with SLE

**Figure 5.**
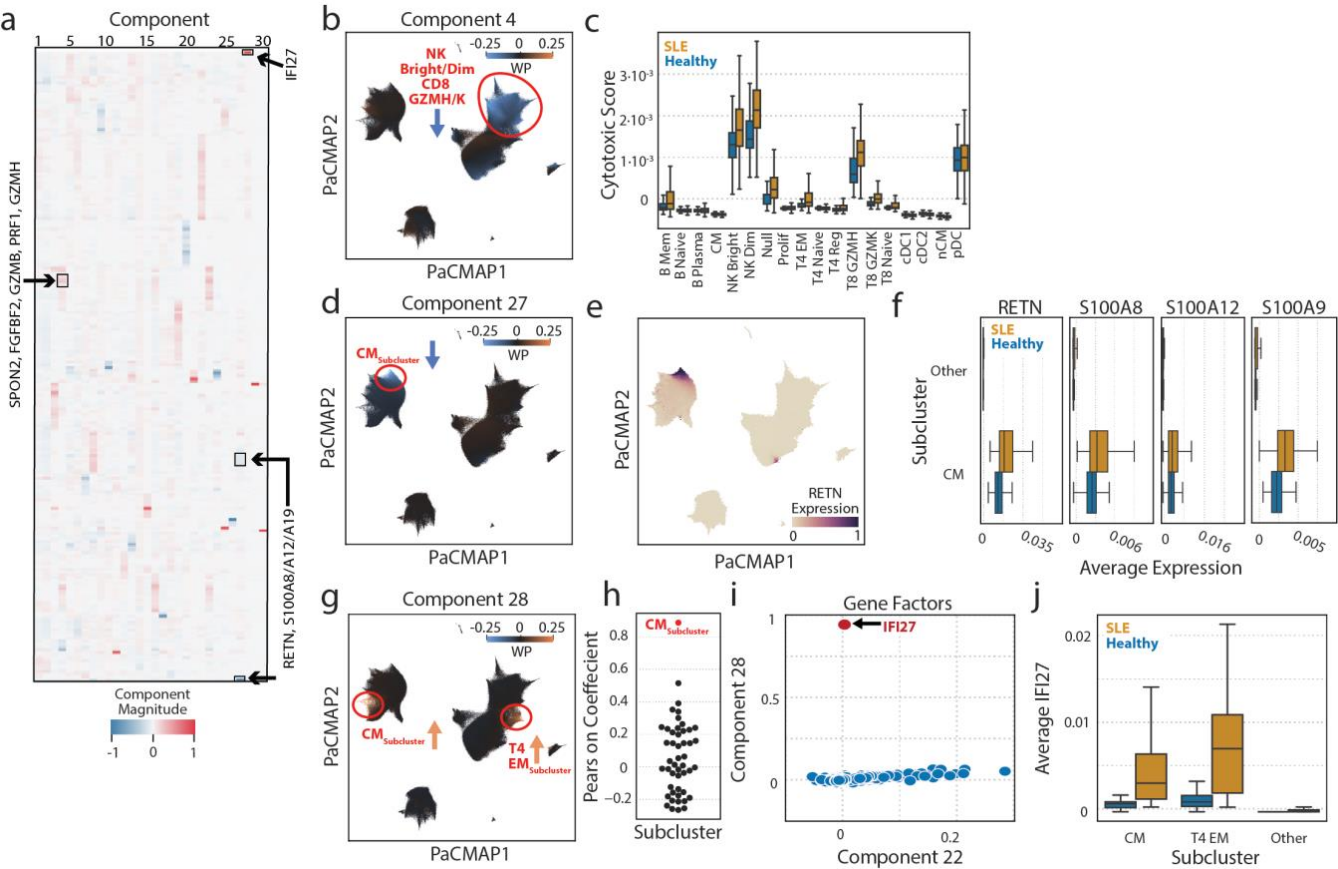
Pf2 identifies high-resolution subpopulation and gene signatures associated with SLE. **(a)** The genes factors matrix with select genes highlighted. **(b, d, g)** PaCMAP of the Pf2 projections, with cells colored by the weighted projections of components (b) 4, (d) 27, and (g) 28. **(c)** Cytotoxic gene score stratified by each cell type. **(d)** Normalized RETN expression of single cells overlayed on PaCMAP embedding **(f, j)** Average (g) RETN, S100A8, S100A12, S100A9, and (k) IFI27 gene expression classified by SLE status and subcluster. **(h)** Pearson correlation coefficient based on the relationship of subcluster cell count per patient against corresponding patient sample weightings of components 28. **(i)** Correlation between component 22 and 28 gene weightings, with IFI27 highlighted.

We next explored other components that yielded novel insights into SLE-associated mechanisms (Fig. 5a). We first inspected component 4 due to its association across several cell types, including GZMH+ CD8+ T cells and several NK populations (Fig. 5b). SPON2, FGFBP2, GZMB, PRF1 and GZMH were among the highly weighted genes associated with component 4 (Fig. 5a, S11). Many of the genes identified by this pattern also overlap with the genes previously used to define a cytotoxic signature for GZMH+ CD8+ T cells^58^. Using the same cytotoxic signature to quantify this gene module, we found that the other immune cell types indeed expressed this signature, including NK Bright, NK Dim, and T8 GZMK+ CD8+ T cells, as identified by component 4 (Fig. 5c). This finding demonstrated that the Pf2 can uncover gene expression changes that are coordinated between cell populations, which would be missed by per-population analyses^62,63^.

In addition to identifying coordinated changes across cell populations, we found that Pf2 identified gene modules in subpopulations of cells that would be missed by a pseudobulk approach. For example, component 27 was highly SLE associated and specifically weighted a small subcluster within the CM population comprising 1% of the total population of cells per patient sample (Fig. 5e). The gene expression for the most weighted gene, RETN, directly coincides with the cells identified by component 27 (Fig. 5a, 5d, 5f). In addition to RETN, the genes S100A8, S100A12, and S100A9 were highly weighted, which others have identified these as relevant to IL-6 signaling and activation of toll-like receptors 4 and 7 in SLE patients, prolonging inflammation (Fig. 5a, S11) ^64–67^. The CM subpopulation is distinct in its shared expression of RETN, S100A8, S100A12, and S100A9 as compared to all other cells (Fig. 5g).

Furthermore, Pf2 can identify subpopulations of cells between cell types with similar expression signatures. The SLE-associated component 28 is highly specific to CM and CD4+ EM subpopulations (Fig. 5h). The component 28 sample weightings were most correlated with the cell abundance of the CM subpopulation and component 28 identified many IFN genes including IFI27, IFI6, ISG15, IFITM3 and IFI44L (Fig 5c). Although some of these genes are highly weighted in other components like in component 22, component 28 strongly weights IFI27 which is specific to these CM and CD4+ EM subpopulations (Fig. 5i, S11). As expected, the subclusters for both CM and CD4+ EM have a higher average expression of IFI27 in SLE patient samples (Fig. 5j). Thus, one can conclude there are subclusters of both CM and CD4+ effector memory cells that share this unique expression of IFI27, along with other type 1 interferon genes. Indeed, IFI27 has been suggested as a potential biomarker for SLE in CD4+ T cells and monocytes^61,68–70^. However, our finding hints that not all cells categorized as CD4+ EM and CM contribute to this unique expression gene module.

## DISCUSSION

Here, we demonstrated that Pf2 can improve investigation and understanding of single cell experiments across conditions. By accounting for the lack of alignment in cells between conditions, single-cell data can be factored with tensor decomposition, enabling the benefits of these linear methods. Because Pf2 is an unsupervised data reduction method, no prior information is required about cell identity or the types of experimental conditions, making Pf2 unbiased as compared to other models that utilize a reference atlas. In both studies above, Pf2 identified transcriptional effects that were shared in all conditions or specific to a subset and isolated this variation to populations of cells. Overall, these results show how a tensor-based approach to single-cell data leads to unique insights that cannot be identified by dimensionality reduction in matrix form or standard pseudobulk analyses.

The advantage of analyzing single cell experiments across conditions with Pf2 was first demonstrated for the exploratory analysis of drug perturbations. While earlier analysis demonstrated the ability to identify perturbed populations at a high level, distinguishing T cells and monocytes, the Pf2 components provide quantitative and qualitative information about perturbations integratively and at high resolution. Despite not requiring cell annotations, Pf2 isolated rare subpopulations, such as pDCs, and their canonical markers including FXYD2 and RARRES2/chemerin^71^. It also distinguished several qualitatively distinct glucocorticoid responses, including conversion of cell identity to myeloid derived suppressor cells and variation in the quality of responses across monocyte subsets^72^. While prior analysis localized Alprostadil effects to monocytes overall, Pf2 demonstrated that DCs were most affected by Alprostadil treatment and located which genes had a shared pro-angiogenesis signature.

The strengths of Pf2 were especially evident in more complex single-cell transcriptomic data from SLE patients. While earlier analysis relied on pseudobulk analysis, we demonstrated that fully considering the single cell data improves one’s ability to predict SLE status and determine which variation patterns are most relevant to the disease. Pf2 confirmed known alterations in naïve T cell and monocyte abundances, while also recovering differences in CD8+ Naïve T cell maturation, proliferating cells, and cDC1 cell abundances described by others^63,73–75^. Our analysis also uncovered that a coordinated cytotoxic and exhaustion-like GZMH+ expression program extends beyond CD8+ T cells to NK dim, NK bright, B memory cells, GZMK+ CD8+ T cells, and CD4+ T effector memory cells, which have been recently found to be highly significant in SLE disease prorgression^62,76^. Pf2 highlighted gene expression modules in a small subset of monocytes with unique expression of RETN and S100A genes. Other studies have suggested S100A8 and S100A9 as potential biomarkers for SLE and Pf2 suggests that subpopulations of CM are contributing to the pro-inflammatory behavior in SLE^64,65^. Similarly, Pf2 identified upregulated coordinated changes of IFI27 in specific subsets of monocytes and CD4+ T effector memory cells and IFI27 has also been suggested for use as a biomarker^77,78^. Many SLE studies have shown the autoimmune disease is characterized by an overall type 1 IFN-based response, but Pf2 delineated qualitatively distinct IFN signatures according to their gene patterns and cellular localization^79,80^.

Despite the popularity of pseudobulk analysis, these results demonstrate several of the limitations of this approach. One must choose a level of detail at which to annotate the cells, with the ultimate results and even basic features such as statistical power affected by the number of populations the cells are split into. While many high-abundance cell populations have reliable canonical markers, these results are reliant on accurate cell annotations. Even small fractions of inconsistently annotated cells could lead to artifacts in the gene expression differences. Clustering algorithms are often employed to form populations, which are then annotated. However, there are no guarantees that clustering populations align with their biologically significant counterparts, especially in the presence of technical artifacts between batches of cells^81^. Finally, cells exist in a continuum of potentially overlapping identities, which are impossible to completely capture as distinct clusters. For instance, naïve, effector, and memory populations exist across CD4, CD8, and NK cell populations. Consequently, it is not possible to form accurate and discrete classes of cells. Instead, Pf2 offers an alternative perspective of cell “eigen-states”, which are multivariate and capture these distinct types of identities.

Both the strengths and limitations of Pf2 can be understood from observing that it is a linear dimensionality reduction method. The method does not attempt to identify or correct for batch effects or other technical artifacts. Indeed, while there have been many attempts to understand and correct for these issues, there is a lack of agreement as to the extent of technical artifacts within single cell RNA-seq measurements, and many study designs do not enable a definitive line to be drawn between biological or technical variation^47,82^. Instead, Pf2 can be used to identify patterns that are associated with batches; for example, the condition factors could be used to identify which patterns are associated with batches. By identifying component patterns, these gene expression alterations can be isolated and inspected. As a factorization method, defining the optimal number of components can be difficult. Cross-validation or other strategies may help to identify the correct number of components when there is not an association prediction or other task with which to evaluate the resulting factors^41,83^. While many components have been necessary to explain substantial fractions of data variance, this enables one to sift through component patterns with predictive modeling, as we have done here.

An important strength of Pf2 is that it provides a foundational framework that can be expanded across various tasks. Additional constraints on the factors, such as nonnegativity, can enhance interpretation of the results^84^. The Pf2 algorithm can be altered to be a supervised factorization approach, leveraging information about each sample to better identify patterns that correspond to features on the cell or condition/sample scale^85^. Pf2 is well-suited to analyze single-omic analysis, but can be broadened to perform coupled decomposition for multi-omic data, such as combining transcriptomics with proteomics, to identify associative patterns across conditions^86^. With modest extension, such as constraints to account for spatial localization, Pf2 could be extended for identifying gene expression patterns in spatial measurements^45,87^.

In summary, we find that Pf2 is especially effective for modeling and exploring multi-condition single cell studies in both perturbational experiments and in an autoimmune cohort. Pf2 localizes variation to specific samples, genes, and populations of cells while enabling interpretation beyond what is possible with non-linear approaches such as autoencoders^88^. As the scale, capabilities, and questions asked using single cell techniques expands, fundamentally new approaches such as Pf2 that are scalable, easily interpretable, and unsupervised will be needed to understand how single cells coordinate their function within and across tissues^31^.

## MATERIALS AND METHODS

### Pf2 Model

In single-cell transcriptomics, a different set of cells is profiled for each condition, and the number of cells profiled in each condition may vary. This precludes the direct arrangement of these measurements into a single tensor, and thus also the direct use of many existing tensor-based decomposition methods, such as PARAFAC^89^. This data would instead be better represented as a length−I list, $X_{\mathrm{sc}}$, of second-order tensors, $X_{\text{sc,i}}\in \;{\mathbb{R}}^{J_{sc,i\times K}}$, i = 1, 2, … , I, where I is the number of conditions, $J_{sc,i}$ is the number of cells profiled in condition i, and K is the number of genes. Pf2 explicitly handles data of this form, where one axis (in this case the cell axis) varies across another (the condition axis). Here, we use the direct fitting algorithm originally proposed by Kiers et al.^41^, to fit the Pf2 model. We also use all-at-once Nesterov-like acceleration for Pf2 as previously described^90^, with extrapolation step size determination parameters of γ = 1.1, $\bar{\gamma }\;=\;1.03$, η = 1.5, $\beta_{0}=\;0.05$,$\bar{{\text{β}}_{0}}\;=\;1$.

In fitting the Pf2 model, we seek

$$\underset{A,B,C,P}{\arg\min}{\sum_{i=1}^{I}\left\|{\text{X}}_{\mathrm{sc,i}}\;-\;P_{i}Bdiag\left(a_{i}\right)C^{T}\right\|}^{2}\;\mathrm{subject}P_{i}^{T}P_{i}\;=\;I\;\forall \;i$$

where $P\;=\;P_{i}\;\in \;{\mathbb{R}}^{J_{sc,i}\times R}$, i = 1, 2, … , I are the projection matrices, where R is the rank of the Pf2 decomposition; $diag\left(a_{i}\right)$ is the diagonal matrix whose nonzero entries are equal to the $i^{\mathrm{th}}$ row of $A\;\in \;{\mathbb{R}}^{I\times R}$ the condition factor matrix; $B\;\in \;{\mathbb{R}}^{R\times R}$ is the cell eigen-state factor matrix; $C\;\in \;{\mathbb{R}}^{K\times R}$ is the gene factor matrix; and I is the identity matrix^41^. Alternating least-squares is used, whereby P and the factor matrices, A, B, and C, are alternately fit. First, the projection matrices are updated as the product of the first R left and right singular vectors from the SVD (we used randomized SVD^91^) of $\mathrm{Bdiag}\left(a_{i}\right)C^{T}X_{sc,i}^{T}$^41^. The factor matrices are then updated as

$$\underset{A,B,C}{\arg\min}{\sum_{i=1}^{I}\left\|{\text{X}}_{\mathrm{sc,i}}\;-\;P_{i}Bdiag\left(a_{i}\right)C^{T}\right\|}^{2}\;=\underset{A,B,C}{\arg\min}{\sum_{i=1}^{I}\left\|{\text{P}}_{i}^{T}{\text{X}}_{\mathrm{sc,i}}\;-\;Bdiag\left(a_{i}\right)C^{T}\right\|}^{2}\;$$

which is equivalent to running PARAFAC^89^ on the single, aligned, third-order tensor whose $i^{th}$ slice along the first mode is equal to ${\text{P}}_{i}^{T}{\text{X}}_{\mathrm{sc,i}}$ ^41^. We used the TensorLy implementation of PARAFAC^92^. We used 20 iterations of PARAFAC per Pf2 iteration and used the TensorLy package defaults for the other PARAFAC parameters. We terminated the Pf2 fitting algorithm once either 200 iterations had elapsed or the difference in R2X between iterations was less than 10^−6^.

### Parameter Initialization

Parameter initialization was performed by using the SVD of the matrix-flattened data, as previously described^41^. Each of ${\text{X}}_{\mathrm{sc,i}}$ was concatenated along the cell dimension to form a cell by gene matrix containing cells from every condition. Randomized SVD^91^ was performed on this matrix and the first R right singular vectors were used to initialize B. A was filled with ones and C was initialized to the identity matrix.

### Post-Factorization Alignment

Like PCA, CP-based decomposition component patterns are made up of the product of vector weights for each dimension in the data. CP-based decompositions generally provide a unique solution, aside from three indeterminacies: the sign of the factors, scaling between factors, and ordering of the components. We therefore applied rules to yield consistent factorization results, helping with the interpretation of the results. To address the scaling indeterminacy, we normalized each factor matrix to have components of unit scale. The sign indeterminacy was then resolved by making the average of each component within the conditions and cell eigen-states factors positive, moving any negative signs to the gene factors.

Pf2 factors are order indeterminant, where the order of each component can be arbitrarily arranged. 𝐴, the conditions factor, was used to arrange components according to their Gini coefficient of association across the conditions, so that the first components were generally found to be associated with all the conditions, while the later components were generally condition specific.

### Reconstruction Error (R2X)

We used the metric reconstruction error (R2X), or the percentage of variance explained by the model, to assess the model fit. First, the Frobenius norm squared of the difference is calculated between the data $\left(X_{data}\right)$ and the reconstruction based of the factors and projections $\left(X_{Pf2}\right)$. This difference is normalized by the Frobenius norm of the data and subtracted from 1 to result in a range from 0 to 1, where 1 represents 100% variance explained.


$$R2X\;=\;1\;-\;\frac{{\left\|X_{data}\;-\;X_{Pf2}\right\|}^{2}}{{\left\|X_{data}\right\|}^{2}}$$

### Factor Match Score (FMS)

The factor match score (FMS) measures the similarity between two sets of factors^45^. Two FMS strategies were implemented. First, to determine how the size of the data influences the factors, the FMS was compared between Pf2 factorizations with the same number of components on the entire dataset and with various fractions of the cells removed. Second, to determine the stability of the factorization at varying numbers of components, cells were bootstrapped to create a dataset of the same size. The FMS was then calculated for the original dataset and the reconstructed dataset at various components. Thus, after 2 unique Pf2 factorizations are applied in both cases, the FMS compares the conditions (A) and genes (B) factor matrices. The eigen-states factors vary in what cells they represent, so they are not included in calculating the FMS. An FMS of 1 indicates complete similarity. A linear sum assignment is also applied because a decomposition can have the same components, but in a different order.


$$FMS\;=\;\sum_{r=1}^{R}\left(1\;-\;\frac{w_{i}w_{i}}{\max\left(w_{i}w_{i}\right)}\right)\cdot \frac{A_{i}^{T}A_{j}}{\left\|A_{i}\right\|\left\|A_{j}\right\|}\cdot \frac{B_{i}^{T}B_{j}}{\left\|B_{i}\right\|\left\|B_{j}\right\|}\;w\;=\;\left\|A\right\|\cdot \left\|B\right\|$$

### Cell type abundance change experiments

To stimulate experiments where a cell type is not present, we removed cells labeled as a specific cell type and determined if Pf2 could identify this change. First, Pf2 was applied to all the cells and conditions of a dataset. Then, the cells of X cell type were removed for a specific experiment, Y, and Pf2 was applied to this cell type-perturbed dataset. After comparing the canonical genes highly weighted for the gene factors for both analyses, the conditions’ component corresponding to canonical markers of the cell type were identified and compared between the two cases. To stimulate an experiment with fewer cells of a certain cell type than other conditions, we subsampled the given cell type by the indicated amount, rather than removing all cells.

### Data Normalization, Processing, Cell Annotation, and Visualization

#### Perturbation Study Analysis

##### Data Normalization and Processing:

The single-cell gene expression was obtained from Chen et al^13^. The first step before normalization was removing doublets with the doubletdetection package applied to the raw RNA counts matrix^93^. Cells with a doublet probability of 0.5 or more were removed from the dataset.

Once doublets were removed, the data was normalized. Genes with less than 0.01 mean raw expression across all cells were removed. Each cell was then normalized by read depth using CPM normalization. Next, each gene was scaled according to its sum. Finally, the data was log transformed via the Delta method,

$$g\left(y\right)\;=\;\log\left(y\;+\;100\right)\;$$

where $\text{g}\left(\text{y}\right)\;$ is the transformed gene and y is the pre-transformed gene^94^. Finally, each gene was mean-centered. The final size of the dataset included 29433 cells and 9461 genes. The data was then fit using the Pf2 model with 20 components as described above. PaCMAP was used on the concatenated matrix of the projections across all conditions to visualize the cell-to-cell variation and embed cells into low-dimensional space, using default parameters of n_neighbors=10, MN=0.5, and FP_ratio=2.046.

##### Cell Type Annotation:

Lower-resolution cellular clusters were defined by Leiden clustering of the Pf2 projections, resulting in 6 Leiden clusters, with parameters n_neighbors=10 for the nearest neighbors distance and resolution=2.5 for the Leiden algorithm^95^. To provide each cluster with a cell type annotation, genes expressed uniquely within each cluster were found using differential expression analysis by the dispersion-based method with parameters of min_mean=0.005, max_mean=10, min_disp=0.5^96^. These genes were used to annotate each cluster based on their overlap with previously reported marker genes^13^. After the lower-resolution assignments, a finer-grained annotation was performed based on each cluster’s expression of a new set of marker genes (Table S4).

#### SLE Analysis

##### Data Normalization and Processing:

Previous analysis used batch corrected data^58^. To prevent potential sources of bias, we used the raw RNA counts matrix after doublet analysis by the original authors. The same normalization pipeline was used as above, except genes with less than 0.1 mean raw expression across all cells were removed. The final size of the dataset was 1,263,673 cells across all conditions and 2,161 genes. The data was then fit using the Pf2 model with 30 components as described above.

##### Cell Type Annotation:

Once again, a similar process was used to assign lower and higher resolution cell types as above. Nearest neighbors distance with n_neighbors=10 and resolution=3 for the Leiden algorithm was used to detect 47 Leiden populations. Low and high-resolution cell types were assigned based on a set of marker genes included in Perez et al (Table S4)^58^.

### Logistic Regression

Using scikit learn, we used a cross-validation strategy of training on one process batch and then predicting the three remaining batches^97^. The regularized logistic regression with a L1 penalty was trained on the Pf2 condition factors. Solving was performed with the Stochastic Average Gradient (SAGA) solver, a tolerance of 1.0 × 10^-4^, and a maximum iteration of 10,000^98^. Both the prediction accuracy and ROC AUC were used as a metric to measure the ability to associate the factors with SLE status. Regularization strength was fit by grid search using the cross-validation implementation described above.

### Principal Components Analysis (PCA)

Principal components analysis (PCA) was performed using scikit learn^97^. The data was “flattened” into a two-dimensional matrix of all cells by genes. The number of components was set to match Pf2.

### Gene Set Enrichment Analysis

Genes corresponding to the 30 most positively and negatively weighted genes for component were submitted to ToppGene and significantly (FDR < 0.05) enriched biological processes were retained^99^.

## Highlights:

- PARAFAC2 enables tensor-based analysis of single-cell experiments across conditions.
- PARAFAC2 separates condition-specific effects from cell-to-cell variation.
- PARAFAC2 provides intuitive isolation of patterns into condition-, cell-, and gene-specific patterns.

## Data Sharing Plan:

All analysis was implemented in Python v3.12 and can be found at https://github.com/meyer-lab/scCP, release 1.0, along with the experimental data.
